# Association between Gout and Gastric Cancer: A Nested Case–Control Study Using a National Health Sample Cohort

**DOI:** 10.3390/jpm14030229

**Published:** 2024-02-21

**Authors:** Mi Jung Kwon, Kyeong Min Han, Ji Hee Kim, Joo-Hee Kim, Min-Jeong Kim, Nan Young Kim, Hyo Geun Choi, Ho Suk Kang

**Affiliations:** 1 Department of Pathology, Hallym University Sacred Heart Hospital, Hallym University College of Medicine, Anyang 14068, Republic of Korea; mulank99@hallym.or.kr; 2 Hallym Data Science Laboratory, Hallym University College of Medicine, Anyang 14068, Republic of Korea; 3 Department of Neurosurgery, Hallym University Sacred Heart Hospital, Hallym University College of Medicine, Anyang 14068, Republic of Korea; 4 Division of Pulmonary, Allergy, and Critical Care Medicine, Department of Medicine, Hallym University Sacred Heart Hospital, Hallym University College of Medicine, Anyang 14068, Republic of Korea; 5 Department of Radiology, Hallym University Sacred Heart Hospital, Hallym University College of Medicine, Anyang 14068, Republic of Korea; 6 Hallym Institute of Translational Genomics and Bioinformatics, Hallym University Medical Center, Anyang 14068 , Republic of Korea; 7 Suseo Seoul E.N.T. Clinic and MD Analytics, Seoul 06349, Republic of Korea; 8 Department of Internal Medicine, Hallym University Sacred Heart Hospital, Hallym University College of Medicine, Anyang 14068, Republic of Korea

**Keywords:** gastric cancer, gout, national healthcare data, nested case–control study

## Abstract

Given the global significance of gout and gastric cancer (GC) as major health problems with interrelated impacts, we examined the development of GC in Korean patients with gout. We conducted a nested case–control study using data from 10,174 GC patients and 40,696 control patients from the Korean National Health Insurance Service-National Sample Cohort database. Propensity score matching (1:4) with propensity score overlap-weighted adjustment was used to reduce selection bias and estimate the odds ratio (OR) and 95% confidence intervals (CIs) for the association between gout and GC. An adjusted OR for GC was not significantly higher in patients with gout than in control patients (1.02; 95% CI, 0.93–1.12; *p* = 0.652). Additionally, no association between gout and GC was observed in subgroup analyses such as sex, age, level of income, region of residence, or Charlson Comorbidity Index score. In conclusion, these results suggest that gout is not a significant independent risk factor for GC among the Korean population. Additional investigation is required to establish a causal association between gout and GC, and to generalize these results to general populations.

## 1. Introduction

Gastric cancer (GC) was the fifth most common malignant tumor worldwide in 2020, with approximately 1.1 million new cases. It is also the fourth leading cause of cancer-related mortalities, with approximately 800,000 deaths [1]. In 2021, Korea recorded the world’s second highest incidence of GC at 51.7 cases per 100,000 people yearly; it is the fifth most widespread cancer among all cancers and has become the most prevalent cancer in males aged 35–64 years [2]. Therefore, identifying important modifiable risk factors responsible for the increased incidence of GC is urgently required.

Gout is the most common type of inflammatory arthritis. It occurs in patients with hyperuricemia and refers to a disease caused by the deposition of uric acid crystals. Among various rheumatic diseases, gout is a disease whose cause is most clearly known, and its treatment is standardized. However, its incidence rate is continuously increasing. According to the Global Burden of Disease Study 2019, the global incidence of gout has increased by 63.44% over the past 20 years, resulting in a 51.12% increase in the number of years lived with a disability [3]. Gout is associated with various systemic and metabolic diseases, including cardiovascular disease, chronic kidney disease, neurodegenerative diseases, diabetes, osteoporosis, and metabolic syndrome [4]. Two recent nationwide population-based studies have shown that patients with gout have an increased overall risk of cancer [5,6]; moreover, gout has been speculated to play an important role in carcinogenesis due to the pro- and antioxidant properties of uric acid [7]. However, the risk of specific cancer types, including GC, remains controversial in patients with gout. Several studies have shown a significant positive relationship between gout and GC [5,6,8], whereas others have reported no such association [9,10].

We hypothesized that patient–demographic factors, such as sex, age, level of income, region of residence, and underlying comorbidities, could affect the association between gout and the likelihood of GC occurrence in the Korean population. Therefore, to explore this hypothesis, we conducted a nested case–control study with inclusive subgroup analyses using Korean public healthcare data to examine the potential effects of gout on GC occurrence.

## 2. Materials and Methods

### 2.1. Study Population

This study protocol was granted by the Hallym University Ethics Committee (approval number: 2019-10-023). The Institutional Review Board waived the requirement for written informed consent due to the use of de-identified secondary data. This study used data from the Korean National Health Insurance Service-National Sample Cohort (KNHIS-NSC), which comprises 1,137,861 patients and 219,673,817 medical claim codes documented between January 2002 and December 2019 [11]. The KNHIS-NSC is a population-based cohort established by Korea’s National Health Insurance Service, aiming to provide public health researchers and policymakers with representative and useful information on individuals’ health insurance use and health inspection. A representative sample cohort comprising 1,025,340 participants, representing 2.2% of the total eligible Korean population in 2002, was randomly selected and followed up for 18 years until 2019, unless they lost eligibility due to death or emigration. In the KNHIS-NSC, 1,476 strata were created using 18 age groups (infants < 1 year, ages 1–4 years, 5-year age groups between 5 and 79 years, and ≥80 years), 2 sex groups (male and female), and 41 patient’s income level groups (upper 20 percentiles for insured employees, lower 20 percentiles for insured self-employed individuals, and the lowest level of income for medical aid beneficiaries). Subsequently, systematic sampling was conducted within 1,476 strata after arranging population data by the value of total annual medical costs and keeping a sampling rate of 2.2%. The cohort contains four databases on participants’ insurance eligibility (such as participant’s identity, sex, region of residence, type of health insurance, level of income, birth, and death), medical care institutions, medical treatments (participant’s electronic medical treatment bills, bill details, details of diseases, and details of prescription), and general health examinations. Participants’ residential information was collected mainly through work addresses until 2005, and residential addresses were recorded from 2006 onwards [11]. The KNHIS-NSC database adheres to the 10th edition of the International Classification of Diseases’ (ICD-10) codes for the standardization of disease diagnoses and organization of healthcare information. Further information about the KNHIS-NSC can be found in other relevant literature [12].

### 2.2. Definition of Gout

To confirm the accuracy of the analysis by removing false-positive cases, gout cases were defined as patients who visited the clinic or hospital with a diagnosis of gout (ICD-10: K05.3) ≥ 2 times [13].

### 2.3. Definition of Gastric Cancer

GC cases were identified using the specific ICD-10 code C16. To ensure the inclusion of individuals with cancer by removing false-positive cancer cases, participants were further selected based on the existence of special claims codes for diagnosed cancer as follows: V193 or V194. In Korea, these special claims codes serve a dual purpose by indicating the diagnosis of severe cancer and marking the individual’s eligibility for reduced healthcare expenses. We enrolled the GC and control groups from the KNHIS-NSC data since 2005 because these special claim codes have been granted by the government since that year.

### 2.4. Patient Selection

**Figure 1:**
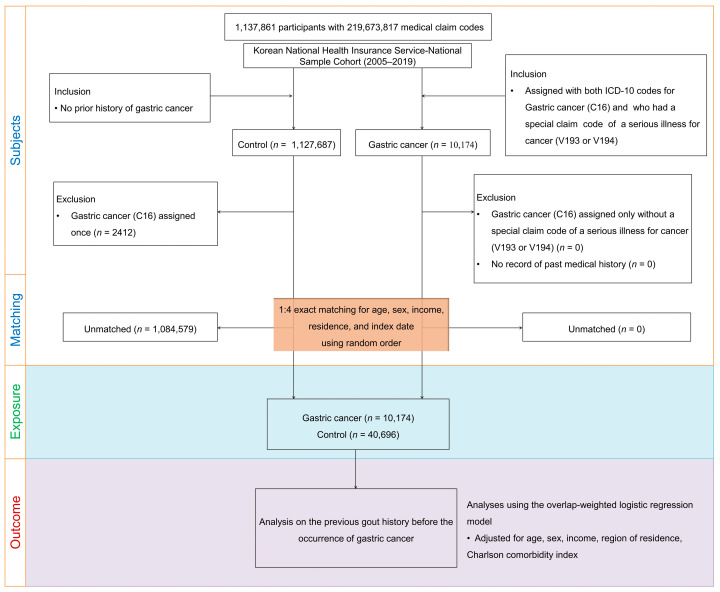
A schematic flowchart of the patient selection method. Among the initial pool of 1,127,681 participants, 10,174 patients with gastric cancer were carefully matched with 40,696 control patients based on factors such as age, sex, level of income, and region of residence.

A nested case–control study design was used to investigate the correlation between the patient’s demographic characteristics, exposure status, and outcomes. We selected 10,174 patients with GC from 1,137,861 patients with 219,673,817 medical claims codes between 2005 and 2019. The control group included patients who were not diagnosed with GC between 2005 and 2019 (*n* = 1,127,687). Control patients diagnosed with GC at least once (*n* = 2412) were excluded. To further strengthen the study’s accuracy and minimize bias, we employed a 1:4 matching technique for each patient with GC, aligning them with control patients based on age, sex, level of income, and region of residence. The control patients were randomly selected to reduce selection bias. The first GC treatment date was set as the index date for each patient with GC, while the index date for control patients was set to that of the matched patients with GC. Therefore, each matched patient with GC, and the control patient had an identical index date. Overall, 1,084,579 control participants were excluded during the matching procedure; finally, a total of 40,696 control patients were included in this study (Figure 1).

### 2.5. Covariates

Patients were classified into 10 age groups at 5-year intervals and further categorized into five levels of income categories, from levels 1 (lowest income) to 5 (highest income). Regions of residence were categorized into 16 administrative districts and further categorized into rural or urban areas [14]. The Charlson Comorbidity Index (CCI) was used to evaluate the disease burden of comorbidities, assigning a sum score ranging from 0 to 29 based on 17 potential comorbidities [15]. CCI scores for both the GC and control groups were collected before the index date between January 2005 and December 2019. However, we excluded cancers from the CCI score to inspect the potential effect of other comorbidities on GC occurrence [15].

### 2.6. Statistical Analysis

When deciding on the GC and control groups, 1:4 propensity score matching was performed based on age, sex, region of residence, and level of income. We compared the general characteristics of patients with GC to those of the control patients using standardized differences. An absolute standardized difference of < 0.20 indicates good balance for a particular covariate, and logistic regression was performed to adjust for covariates for standardized differences of >0.01 [16]. We employed propensity score overlap weighting to maintain the exact balance and to adjust the precision to reduce the probability of intergroup bias. The patients with GC were weighted using the probability of the propensity score, whereas the control participants were weighted with the probability of 1- propensity score, ranging from 0 to 1 [17,18,19]. To explore the odds ratios (ORs) and their corresponding 95% confidence intervals (CIs) for a history of gout before the index date and GC occurrence, a propensity score overlap-weighted multivariable logistic regression analysis was performed for age, sex, region of residence, level of income, CCI score, and history of gout. Therefore, “before (unadjusted)” and “after (adjusted for age, sex, level of income, region of residence, CCI score, and history of gout)” propensity score overlap-weighted adjustment models were used for these analyses. Subgroup analyses were performed by all covariates. Two-tailed analyses were implemented, and significance was defined as *p* values less than 0.05. SAS version 9.4 (SAS Institute Inc., Cary, NC, USA) was used for statistical analyses.

## 3. Results

### 3.1. Baseline Characteristics of the Study Patients

This study included 10,174 patients with GC, 40,696 control patients, and an equivalent number of age, sex, region of residence, and level of income matched comparison participants (Table 1). Before applying the propensity score overlap-weighted adjustment model, age, sex, region of residence, and level of income had an absolute standardized mean difference of 0.00, representing no difference between the GC and control groups. However, other covariates, such as CCI scores and history of gout before the index date, were imbalanced between the GC and control groups (standardized differences of 0.67 and 0.02, respectively). After the overlap-weighted adjustment process, the standardized difference became negligible, achieving balance across all characteristics, including CCI scores and history of gout (standardized difference of 0.00 and 0.00, respectively).

### 3.2. Relationship between Gout and Gastric Cancer

The crude and adjusted ORs for GC incidence in patients with gout are shown in Table 2. The ORs for GC incidence did not significantly differ between gout and control groups in both crude and overlap-weighted adjusted models (crude OR, 1.09; 95% CI, 0.98–1.23; *p* = 0.127; adjusted OR, 1.02; 95% CI, 0.93–1.12; *p* = 0.652).

### 3.3. Subgroup Analysis

A comprehensive subgroup analysis examining variables, such as age, sex, level of income, region of residence, and CCI scores was used to further investigate the relationship between gout and GC (Table 2). In the subgroup analysis of demographic characteristics, male (adjusted OR, 1.01; 95% CI, 0.92–1.11; *p* = 0.842), female (adjusted OR, 1.16; 95% CI, 0.87–1.54; *p* = 0.306), age < 65 years (adjusted OR, 1.04; 95% CI, 0.90–1.20; *p* = 0.639), age ≥ 65 years (adjusted OR, 1.02; 95% CI, 0.90–1.15; *p* = 0.774), low income (adjusted OR, 0.98; 95% CI, 0.85–1.12; *p* = 0.74), high income (adjusted OR, 1.06; 95% CI, 0.93–1.20; *p* = 0.375), urban resident (adjusted OR, 1.11; 95% CI, 0.96–1.28; *p* = 0.146), and rural resident (adjusted OR, 0.96; 95% CI, 0.85–1.08; *p* = 0.512) groups showed no significant association between gout and GC occurrence. Additionally, subgroup analyses regarding CCI score (CCI scores of 0 (adjusted OR, 1.10; 95% CI, 0.96–1.26; *p* = 0.185), 1 (adjusted OR, 0.88; 95% CI, 0.70–1.12; *p* = 0.292), and ≥ 2 (adjusted OR, 1.04; 95% CI, 0.89–1.21; *p* = 0.644)) also showed no significant association between gout and GC development.

## 4. Discussion

This study showed that, compared with the control group, subjects with gout may not be at risk for GC using the propensity score overlap-weighted multivariable logistic regression analysis model adjusted for comorbidities, socioeconomic variables, and demographic characteristics. Moreover, differences in the risk association depending on specific demographic factors were not observed. This finding suggests that gout is not likely a significant independent risk factor for GC in the general population.

Uric acid is a powerful antioxidant at physiological concentrations; however, it shows pro-oxidant activity at high intracellular concentrations. Therefore, uric acid may play an important role in carcinogenesis processes [20,21]. Indeed, both hyperuricemia and hypouricemia have been correlated with increased cancer risk [7]. Many cohort studies have shown a U-shaped association between serum uric acid levels and increased cancer mortality [22,23,24]. Additionally, medications frequently used for gout, such as allopurinol or colchicine, may exert anti-cancer effects by mitigating oxidative stress [25] or disrupting microtubules [26,27]. Therefore, determining the effects of gout on cancer in the real world is challenging, and identifying this relationship and determining the cause through epidemiological studies and experimental procedures, respectively, is necessary.

Only five epidemiological studies have explored the impact of gout on GC incidence. Our findings align with those of nationwide database studies conducted in Sweden and Taiwan. A Swedish study of 16,857 patients with gout showed that the standardized incidence ratios of GC were 0.97 (95% CI, 0.71–1.30) and 1.11 (95% CI, 0.65–1.78) for males and females, respectively [9]. In a Taiwan study targeting 25,943 patients with gout, individuals with gout had a significantly increased incidence of overall cancers compared to the control group (hazard ratio [HR], 1.15, 95% CI, 1.10–1.21); however, GC incidence was not significant (HR, 1.18, 95% CI, 0.97–1.43) [10]. Contrary to the two previous studies, three population-based studies from Taiwan and Korea showed results that contradicted our findings. Two Taiwanese studies, involving 8,408 male patients with gout and males aged 41–55 years, found an association between gout and GC with HRs of 1.80 (95% CI, 1.17–2.75) and 1.71 (95% CI, 1.21–2.39), respectively [5,8]. A Korean study comparing 179,930 patients with gout with the same number in the control group showed that the incidence of overall cancer (adjusted HR, 1.053; (95% CI, 1.031–1.077) and GC (adjusted HR, (95% CI, 1.103; 1.032–1.178, *p* < 0.01) were significantly associated with gout [6]. The reason for these conflicting results is heterogeneity because demographic diversities likely cause large dissimilarities in the original quality of the study group [28]. Additionally, the conflicting results observed in the five existing studies [5,6,8,9,10] could be attributed to several limitations. These studies usually had unfair sample sizes, included groups with uneven demographic characteristics, such as sex, age, and socioeconomic status, and focused on specific age ranges or sexes [5,6,8]. Some of the studies also lacked control groups entirely [8,9,10]. For example, two studies reported a significant link between GC and gout [5,6]. However, these studies had a confounding factor: patients in the gout group had significantly higher rates of underlying diseases (diabetes, high blood pressure, and chronic kidney disease), smoking, alcohol consumption, and obesity than the control group. Although these factors were adjusted in the results, patients with more comorbidities had greater access to medical care, which may have caused the difference in the diagnosis rate of GC. Our study had a substantial sample size, including 10,174 patients with GC and 40,696 control patients from a well-organized nationwide healthcare database (KNHIS-NSC). The KNHIS-NSC database represents approximately 2.2% of the Korean population and was selected by a systematic sampling method using 1,476 strata (18 age, 2 sex, and 41 income level groups). To reduce selection bias within this cohort, we employed a 1:4 matching technique for each patient with GC, aligning them with control patients based on age, sex, level of income, and region of residence. Subsequently, a propensity score overlap-weighted multivariate logistic regression analysis was performed, including the CCI score and history of gout. After applying the overlap-weighted adjustment models, the standardized difference became negligible, achieving balance across all characteristics. Therefore, this study effectively mitigated selection bias by ensuring a well-balanced distribution across key demographic factors, such as age, sex, level of income, region of residence, and CCI score.

To the best of our knowledge, this is the first study to comprehensively analyze the association between GC and gout while exploring how specific demographic factors modulate this risk. Although two previous studies investigated the link between gout and colorectal cancer using similar methods and balanced demographics, our study is the only one to focus on GC and examine such diverse demographic aspects [29,30]. Similarly to our results, these two studies showed that gout may not be an independent significant risk factor for colorectal cancer in the general population. Compared to other organs, the gastrointestinal tract is greatly influenced by symbiotic relationships with gut microbiota. Gut microbiota plays an important role in the development of gastrointestinal cancers, including GC [31,32]. Recent studies have also shown that changes in the composition and metabolism of the gut microbiota are associated with abnormal uric acid breakdown, increased uric acid production, and damage to the intestinal barrier by uric acid [33,34]. Lactobacillus species inhibit uric acid biosynthesis during purine metabolism by degrading inosine and guanosine [35,36]. *Lactobacillus gallinii* fermentation products have a urate-lowering effect, and *Lactobacillus gasseri* strains can reduce intestinal purine absorption [37,38]. Therefore, in gastrointestinal cancers, it must be considered that the specificity of the symbiotic relationship with the microbiome may affect the relationship between cancer and gout, and additional research on this appears to be necessary.

Our study is the only one that focused on GC rather than overall cancer and conducted a subgroup analysis according to age, sex, level of income, region of residence, and CCI score. The most important finding of our study was that Korean patients with gout may not have an increased risk of developing GC, regardless of age and sex. However, this study also has some limitations. First, the KNHIS-NSC data are inherently limited because they were created using claims data rather than clinical research data. Therefore, patients’ actual disease status and clinical outcomes are not recorded in the data. Records of non-insurance benefits data, such as cosmetic treatments and information for over-the-counter medications, are also not provided since they do not fall within the scope of the claim. Therefore, the KNHIS-NSC database used in this study lacks detailed information regarding factors including gout severity, *Helicobacter pylori* infection status, clinical or pathological stage of GC, histological features of GC, genetic predisposition of GC including family history of cancer, diet, and uric acid levels at baseline and during cancer development. Second, generalization of the study results to other demographic groups outside of South Korea should be made with caution because this study targeted the Korean population and used disease codes from Korean health insurance data. Third, when setting up the control group, we performed 1:4 propensity score matching and corrected the underlying comorbidity, although detection bias due to hospital utilization cannot be completely ruled out. Lastly, conducting follow-up studies is necessary to determine whether the time from diagnosis of gout and the medications used for gout affect GC incidence.

## 5. Conclusions

Our findings showed that patients with gout are not at a higher risk of GC than control patients. Subgroup analyses also showed no association between gout and GC development, regardless of factors including age, sex, level of income level, region of residence, or CCI score. The results of our study, which performed propensity score overlap-weighted multivariate logistic regression analyses using a well-organized nationwide healthcare database, represent that gout may not significantly and independently influence GC risk in the general population. However, further inclusive research is desirable to prove a causal association between gout and GC, and to generalize these results to general populations.

## Figures and Tables

**Table 1:** General characteristics of the patients.

Characteristics		Before PS Overlap-Weighted Adjustment			After PS Overlap-Weighted Adjustment		
		Gastric Cancer	Control	SD	Gastric Cancer	Control	SD
Age (*n*, %)				0.00			0.00
	0–4	N/A	N/A		N/A	N/A	
	5–9	1 (0.01)	4 (0.01)		1 (0.01)	1 (0.01)	
	10–14	3 (0.03)	12 (0.03)		2 (0.02)	2 (0.02)	
	15–19	N/A	N/A		N/A	N/A	
	20–24	1 (0.01)	4 (0.01)		1 (0.01)	1 (0.01)	
	25–29	19 (0.19)	76 (0.19)		13 (0.17)	13 (0.17)	
	30–34	95 (0.93)	380 (0.93)		63 (0.85)	63 (0.85)	
	35–39	205 (2.01)	820 (2.01)		141 (1.92)	141 (1.92)	
	40–44	466 (4.58)	1864 (4.58)		336 (4.57)	336 (4.57)	
	45–49	711 (6.99)	2844 (6.99)		506 (6.89)	506 (6.89)	
	50–54	994 (9.77)	3976 (9.77)		702 (9.56)	702 (9.56)	
	55–59	1197 (11.77)	4788 (11.77)		856 (11.66)	856 (11.66)	
	60–64	1449 (14.24)	5796 (14.24)		1046 (14.24)	1046 (14.24)	
	65–69	1463 (14.38)	5852 (14.38)		1059 (14.43)	1059 (14.43)	
	70–74	1490 (14.65)	5960 (14.65)		1087 (14.80)	1087 (14.80)	
	75–79	1071 (10.53)	4284 (10.53)		787 (10.72)	787 (10.72)	
	80–84	693 (6.81)	2772 (6.81)		509 (6.93)	509 (6.93)	
	85+	316 (3.11)	1264 (3.11)		236 (3.21)	236 (3.21)	
Sex (*n*, %)				0.00			0.00
	Male	6834 (67.17)	27,336 (67.17)		4933 (67.18)	4933 (67.18)	
	Female	3340 (32.83)	13,360 (32.83)		2410 (32.82)	2410 (32.82)	
Income (*n*, %)				0.00			0.00
	1 (lowest)	1959 (19.25)	7836 (19.25)		1404 (19.12)	1404 (19.12)	
	2	1260 (12.38)	5040 (12.38)		899 (12.24)	899 (12.24)	
	3	1621 (15.93)	6484 (15.93)		1169 (15.92)	1169 (15.92)	
	4	2144 (21.07)	8576 (21.07)		1540 (20.97)	1540 (20.97)	
	5 (highest)	3190 (31.35)	12,760 (31.35)		2332 (31.75)	2332 (31.75)	
Region of residence (*n*, %)				0.00			0.00
	Urban	4310 (42.36)	17,240 (42.36)		3111 (42.36)	3111 (42.36)	
	Rural	5864 (57.64)	23,456 (57.64)		4233 (57.64)	4233 (57.64)	
CCI score (Mean, SD)		2.40 (2.70)	0.92 (1.59)	0.67	1.72 (1.89)	1.72 (0.97)	0.00
Gout (*n*, %)		390 (3.83)	1432 (3.52)	0.02	277 (3.78)	272 (3.70)	0.00

Abbreviations: PS, propensity score; CCI, Charlson Comorbidity Index; SD, standardized difference; N/A, not applicable.

**Table 2:** Crude and overlap propensity score weighted odds ratios of gout for gastric cancer and subgroup analyses according to age, sex, level of income, region of residence, and CCI scores.

Characteristics		Number of Patients with GC	Number of Control Patients	Odd Ratios for GC (95% Confidence Interval)			
		(Exposure/Total, %)	(Exposure/Total, %)	Crude	*p*-Value	Overlap-Weighted Model †	*p*-Value
Total patients (*n* = 50,870)							
	Gout	390/10,174 (3.8)	1432/40,696 (3.5)	1.09 (0.98–1.23)	0.127	1.02 (0.93–1.12)	0.652
	Control	9784/10,174 (96.2)	39,264/40,696 (96.5)	1		1	
Age < 65 years (*n* = 25,705)							
	Gout	157/5,141 (3.1)	573/20,564 (2.8)	1.10 (0.92–1.31)	0.302	1.04 (0.90–1.20)	0.639
	Control	4984/5141 (96.9)	19,991/20,564 (97.2)	1		1	
Age ≥ 65 years (*n* = 25,165)							
	Gout	233/5033 (4.6)	859/20,132 (4.3)	1.09 (0.94–1.26)	0.259	1.02 (0.90–1.15)	0.774
	Control	4800/5033 (95.4)	19,273/20,132 (95.7)	1		1	
Male (*n* = 34,170)							
	Gout	348/6,834 (5.1)	1298/27,336 (4.7)	1.08 (0.95–1.22)	0.235	1.01 (0.92–1.11)	0.842
	Control	6486/6834 (94.9)	26,038/27,336 (95.3)	1		1	
Female (*n* = 16,700)							
	Gout	42/3340 (1.3)	134/13,360 (1.0)	1.26 (0.89–1.78)	0.198	1.16 (0.87–1.54)	0.306
	Control	3298/3340 (98.7)	13,226/13,360 (99.0)	1		1	
Low-income (*n* = 24,200)							
	Gout	168/4840 (3.5)	634/19,360 (3.3)	1.06 (0.89–1.26)	0.495	0.98 (0.85–1.12)	0.74
	Control	4672/4840 (96.5)	18,726/19,360 (96.7)	1		1	
High-income (*n* = 26,670)							
	Gout	222/5334 (4.2)	798/21,336 (3.7)	1.12 (0.96–1.30)	0.151	1.06 (0.93–1.20)	0.375
	Control	5112/5334 (95.8)	20,538/21,336 (96.3)	1		1	
Urban resident (*n* = 21,550)							
	Gout	172/4310 (4.0)	605/17,240 (3.5)	1.14 (0.96–1.36)	0.13	1.11 (0.96–1.28)	0.146
	Control	4138/4310 (96.0)	16,635/17,240 (96.5)	1		1	
Rural resident (*n* = 29,320)							
	Gout	218/5864 (3.7)	827/23,456 (3.5)	1.06 (0.91–1.23)	0.472	0.96 (0.85–1.08)	0.512
	Control	5646/5864 (96.3)	22,629/23,456 (96.5)	1		1	
CCI scores = 0 (*n* = 28,141)							
	Gout	110/3480 (3.2)	737/24,661 (3.0)	1.06 (0.86–1.30)	0.577	1.10 (0.96–1.26)	0.185
	Control	3370/3480 (96.8)	23,924/24,661 (97.0)	1		1	
CCI scores = 1 (*n* = 8,958)							
	Gout	62/1990 (3.1)	248/6968 (3.6)	0.87 (0.66–1.16)	0.34	0.88 (0.70–1.12)	0.292
	Control	1928/1990 (96.9)	6720/6968 (96.4)	1		1	
CCI scores ≥ 2 (*n* = 13,771)							
	Gout	218/4704 (4.6)	447/9067 (4.9)	0.94 (0.79–1.11)	0.447	1.04 (0.89–1.21)	0.644
	Control	4486/4704 (95.4)	8620/9067 (95.1)	1		1	

Abbreviations: GC, gastric cancer; CCI, Charlson Comorbidity Index. Significance set at *p* < 0.05. † Adjusted for age, sex, level of income, region of residence, and CCI scores.

## Data Availability

Restrictions apply to the data availability. Data were obtained from the Korean National Health Insurance Sharing Service (NHISS) and are available at https://nhiss.nhis.or.kr (accessed on 25 January 2022) with permission from the NHISS.

## References

[1] Sung H., Ferlay J., Siegel R.L., Laversanne M., Soerjomataram I., Jemal A., Bray F. (2021). Global Cancer Statistics 2020: GLOBOCAN estimates of incidence and mortality worldwide for 36 cancers in 185 countries. CA Cancer J. Clin..

[2] Jung K.W., Won Y.J., Kang M.J., Kong H.J., Im J.S., Seo H.G. (2022). Prediction of cancer incidence and mortality in Korea, 2022. Cancer Res. Treat..

[3] He Q., Mok T.N., Sin T.H., Yin J., Li S., Yin Y., Ming W.K., Feng B. (2023). Global, regional, and national prevalence of gout from 1990 to 2019: Age-period-cohort analysis with future burden prediction. JMIR Public Health Surveill.

[4] Singh J.A., Gaffo A. (2020). Gout epidemiology and comorbidities. Semin. Arthritis Rheum..

[5] Lee J.S., Myung J., Lee H.A., Hong S., Lee C.K., Yoo B., Oh J.S., Kim Y.G. (2021). Risk of cancer in middle-aged patients with gout: A nationwide population-based study in Korea. J. Rheumatol..

[6] Oh Y.J., Lee Y.J., Lee E., Park B., Kwon J.W., Heo J., Moon K.W. (2022). Cancer risk in Korean patients with gout. Korean J. Intern. Med..

[7] Allegrini S., Garcia-Gil M., Pesi R., Camici M., Tozzi M.G. (2022). The good, the bad and the new about uric acid in cancer. Cancers.

[8] Chen C.J., Yen J.H., Chang S.J. (2014). Gout patients have an increased risk of developing most cancers, especially urological cancers. Scand. J. Rheumatol..

[9] Boffetta P., Nordenvall C., Nyrén O., Ye W. (2009). A prospective study of gout and cancer. Eur. J. Cancer Prev..

[10] Kuo C.F., Luo S.F., See L.C., Chou I.J., Fang Y.F., Yu K.H. (2012). Increased risk of cancer among gout patients: A nationwide population study. Jt. Bone Spine.

[11] Lee J., Lee J.S., Park S.H., Shin S.A., Kim K. (2017). Cohort profile: The National Health Insurance Service–National Sample Cohort (NHIS-NSC), South Korea. Int. J. Epidemiol..

[12] Kyoung D.S., Kim H.S. (2022). Understanding and utilizing claim data from the Korean National Health Insurance Service (NHIS) and Health Insurance Review & Assessment (HIRA) database for research. J. Lipid Atheroscler..

[13] Kim J.W., Kwak S.G., Lee H., Kim S.K., Choe J.Y., Park S.H. (2017). Prevalence and incidence of gout in Korea: Data from the national health claims database 2007–2015. Rheumatol. Int..

[14] Kwon M.J., Byun S.H., Kim J.H., Kim J.H., Kim S.H., Kim N.Y., Park H.R., Choi H.G. (2022). Longitudinal follow-up study of the association between statin use and chronic periodontitis using national health screening cohort of Korean population. Sci. Rep..

[15] Quan H., Li B., Couris C.M., Fushimi K., Graham P., Hider P., Januel J.M., Sundararajan V. (2011). Updating and validating the Charlson comorbidity index and score for risk adjustment in hospital discharge abstracts using data from 6 countries. Am. J. Epidemiol..

[16] Austin P.C. (2009). Balance diagnostics for comparing the distribution of baseline covariates between treatment groups in propensity-score matched samples. Stat. Med..

[17] Li F., Morgan K.L., Zaslavsky A.M. (2018). Balancing covariates via propensity score weighting. J. Am. Stat. Assoc..

[18] Slobodnick A., Krasnokutsky S., Lehmann R.A., Keenan R.T., Quach J., Francois F., Pillinger M.H. (2019). Colorectal cancer among gout patients undergoing colonoscopy. J. Clin. Rheumatol..

[19] Thomas L.E., Li F., Pencina M.J. (2020). Overlap weighting: A propensity score method that mimics attributes of a randomized clinical trial. JAMA.

[20] Fini M.A., Elias A., Johnson R.J., Wright R.M. (2012). Contribution of uric acid to cancer risk, recurrence, and mortality. Clin. Transl. Med..

[21] Giebułtowicz J., Wroczyński P., Samolczyk-Wanyura D. (2011). Comparison of antioxidant enzymes activity and the concentration of uric acid in the saliva of patients with oral cavity cancer, odontogenic cysts and healthy subjects. J. Oral Pathol. Med..

[22] Cho S.K., Chang Y., Kim I., Ryu S. (2018). U-Shaped Association Between Serum Uric Acid Level and Risk of Mortality: A Cohort Study. Arthritis Rheumatol..

[23] Huang C.F., Huang J.J., Mi N.N., Lin Y.Y., He Q.S., Lu Y.W., Yue P., Bai B., Zhang J.D., Zhang C. (2020). Associations between serum uric acid and hepatobiliary-pancreatic cancer: A cohort study. World J. Gastroenterol..

[24] Crawley W.T., Jungels C.G., Stenmark K.R., Fini M.A. (2022). U-shaped association of uric acid to overall-cause mortality and its impact on clinical management of hyperuricemia. Redox Biol..

[25] Li Y., Cao T.T., Guo S., Zhong Q., Li C.H., Li Y., Dong L., Zheng S., Wang G., Yin S.F. (2016). Discovery of novel allopurinol derivatives with anticancer activity and attenuated xanthine oxidase inhibition. Molecules.

[26] Cho J.H., Joo Y.H., Shin E.Y., Park E.J., Kim M.S. (2017). Anticancer effects of colchicine on hypopharyngeal cancer. Anticancer Res..

[27] Kumar A., Sharma P.R., Mondhe D.M. (2017). Potential anticancer role of colchicine-based derivatives: An overview. Anticancer Drugs.

[28] Linden A.H., Honekopp J. (2021). Heterogeneity of research results: A new perspective from which to assess and promote progress in psychological science. Perspect. Psychol. Sci..

[29] Chuang J.P., Lee J.C., Leu T.H., Hidajah A.C., Chang Y.H., Li C.Y. (2019). Association of gout and colorectal cancer in Taiwan: A nationwide population-based cohort study. BMJ Open.

[30] Kwon M.J., Han K.M., Kim J.H., Kim J.H., Kim M.J., Kim N.Y., Choi H.G., Kang H.S. (2023). Gout and colorectal cancer likelihood: Insights from a nested case-control study of the Korean population utilizing the Korean National Health Insurance Service-National Sample Cohort. Cancers.

[31] Coker O.O., Dai Z., Nie Y., Zhao G., Cao L., Nakatsu G., Wu W.K., Wong S.H., Chen Z., Sung J.J.Y. (2018). Mucosal microbiome dysbiosis in gastric carcinogenesis. Gut.

[32] Ağagündüz D., Cocozza E., Cemali Ö., Bayazıt A.D., Nanì M.F., Cerqua I., Morgillo F., Saygılı S.K., Berni Canani R., Amero P. (2023). Understanding the role of the gut microbiome in gastrointestinal cancer: A review. Front. Pharmacol..

[33] Tong S., Zhang P., Cheng Q., Chen M., Chen X., Wang Z., Lu X., Wu H. (2022). The role of gut microbiota in gout: Is gut microbiota a potential target for gout treatment. Front. Cell Infect. Microbiol..

[34] Wang Z., Li Y., Liao W., Huang J., Liu Y., Li Z., Tang J. (2022). Gut microbiota remodeling: A promising therapeutic strategy to confront hyperuricemia and gout. Front. Cell Infect. Microbiol..

[35] Azevedo V.F., Kos I.A., Vargas-Santos A.B., da Rocha Castelar Pinheiro G., Dos Santos Paiva E. (2019). Benzbromarone in the treatment of gout. Adv. Rheumatol..

[36] Wu J., Wei Z., Cheng P., Qian C., Xu F., Yang Y., Wang A., Chen W., Sun Z., Lu Y. (2020). Rhein modulates host purine metabolism in intestine through gut microbiota and ameliorates experimental colitis. Theranostics.

[37] Li M., Yang D., Mei L., Yuan L., Xie A., Yuan J. (2014). Screening and characterization of purine nucleoside degrading lactic acid bacteria isolated from Chinese sauerkraut and evaluation of the serum uric acid lowering effect in hyperuricemic rats. PLoS ONE.

[38] Yamada N., Iwamoto C., Kano H., Yamaoka N., Fukuuchi T., Kaneko K., Asami Y. (2016). Evaluation of purine utilization by Lactobacillus gasseri strains with potential to decrease the absorption of food-derived purines in the human intestine. Nucleosides Nucleotides Nucleic Acids.

